# Novel Homozygous MTHFR Variant Causing Homocystinuria: Subtle Phenotypic Clues in Carriers

**DOI:** 10.1016/j.aed.2025.04.007

**Published:** 2025-04-25

**Authors:** Arun Kumar R. Pande, Ashish Jha, Ashwini K. Thakur, Zehra Talat

**Affiliations:** 1 Department of Endocrinology Diabetes and Metabolism, Lucknow Endocrine Diabetes and Thyroid Clinic, Lucknow, Uttar Pradesh, India; 2 Department of Endocrinology, Diabetes and Metabolism, Health City Vistaar, Lucknow, Uttar Pradesh, India; 3 Department of Cardiology, Dr Ram Manohar Lohia Institute of Medical Sciences, Lucknow, Uttar Pradesh, India; 4 Department of Biological Sciences and Bioengineering, The Mehta Family Centre For Engineering in Medicine, Indian Institute of Technology, Kanpur, Uttar Pradesh, India; 5 Department of Biological Sciences and Bioengineering, The Mehta Family Centre For Engineering in Medicine, Indian Institute of Technology, Kanpur, Uttar Pradesh, India

**Keywords:** homocystinuria, tall stature, subtle phenotype in variant carrier, MTHFR

## Abstract

**Background:**

Homocystinuria is a rare metabolic disorder characterized by elevated homocysteine levels due to defects in methionine metabolism.

**Case Report:**

We present a 17-year-old male with tall stature, intellectual disability, and skeletal abnormalities. Elevated plasma homocysteine levels 199.95 μmol/L were noted, which increased to 225.04 μmol/L following pyridoxine therapy, indicating nonresponsiveness. Genetic analysis revealed a novel homozygous missense variant in exon 12 of the MTHFR gene (p.Lys625Thr). Notably, the carrier father exhibited an increased arm span-to-height ratio and raised serum homocysteine level 25.78 μmol/L, a subtle phenotypic and biochemical trait absent in the carrier mother. Management included a low-methionine diet, vitamin supplementation, and initiation of betaine therapy.

**Discussion:**

This case underscores the importance of genetic testing and individualized management in homocystinuria, especially with novel mutations. The observed subtle phenotypic feature in the carrier father highlights the need for comprehensive family evaluations.

**Highlights**

• Unexplained tall stature, intellectual disability, and skeletal abnormalities should prompt early homocysteine measurement to diagnose homocystinuria
• Novel mutations, like the MTHFR (p.Lys625Thr) variant, emphasize the need for genetic analysis in suspected cases
• Pyridoxine nonresponsiveness guides treatment decisions, necessitating dietary modifications and betaine therapy
• Identifying subtle traits in carriers, such as an increased arm span-to-height ratio, enhances early detection and family counseling
• A tailored approach, including dietary and pharmacologic interventions, improves patient outcomes

**Clinical Relevance**

This case highlights the role of genetic testing and comprehensive family assessment in diagnosing and managing homocystinuria. Recognizing subtle phenotypic markers in carriers may facilitate earlier diagnosis and intervention.

**Key messages**

Early homocysteine measurement and genetic testing are crucial for diagnosing homocystinuria, especially in cases of unexplained tall stature. Observing subtle carrier phenotypes, like unusual arm span ratio, aids in early diagnosis.

## Introduction

Tall stature can arise from various underlying conditions, ranging from benign constitutional growth to syndromic or systemic diseases with associated manifestations such as intellectual disability, seizures, skeletal abnormalities, and distinctive facial features.^1^ Homocystinuria, a rare metabolic disorder, is one such condition characterized by elevated homocysteine levels due to genetic defects in methionine metabolism. With a prevalence of approximately 1 in 100 000 births, homocystinuria shows geographic variation, with higher incidences reported in populations such as those in Ireland and Qatar.^2^^,^^3^ This variability underscores the importance of population-specific screening and awareness programs for early diagnosis and management.

In homocystinuria, anthropometric measurements such as increased arm span, arm span-to-height ratio, and upper-to-lower segment ratio are key indicators of skeletal disproportion and marfanoid habitus, which are hallmark features of the condition. Prompt diagnosis and treatment are essential to mitigate symptom progression and prevent long-term disability.^1^

This case report describes a previously unreported homozygous variant in the *MTHFR* gene causing homocystinuria. The subtle clinical presentations observed, particularly in carriers, emphasize the importance of identifying atypical phenotypic features, such as increased arm span-to-height ratio, which can lead to earlier diagnosis and guide family screening efforts. Our findings contribute to expanding the genetic and clinical spectrum of homocystinuria and highlight the significance of comprehensive clinical and biochemical assessments in individuals presenting with tall stature.

## Case Report

We describe the case of a 17-year-old male who has been experiencing abnormally tall stature and reduced cognitive abilities since childhood. His educational achievements are below the expected level for his age, being in the sixth grade, and he has always been significantly taller than his peers. His academic struggles are compounded by a history of seizures commencing at 11 years, for which he has been receiving antiepileptic treatment. Additionally, he had meningitis at the age of 6 but recovered fully. Notably, there is a history of second-degree consanguinity in his family, with a similar tall stature observed in his younger brother. He also had a history of delayed motor and psychomotor milestones.

**Fig. 1:**
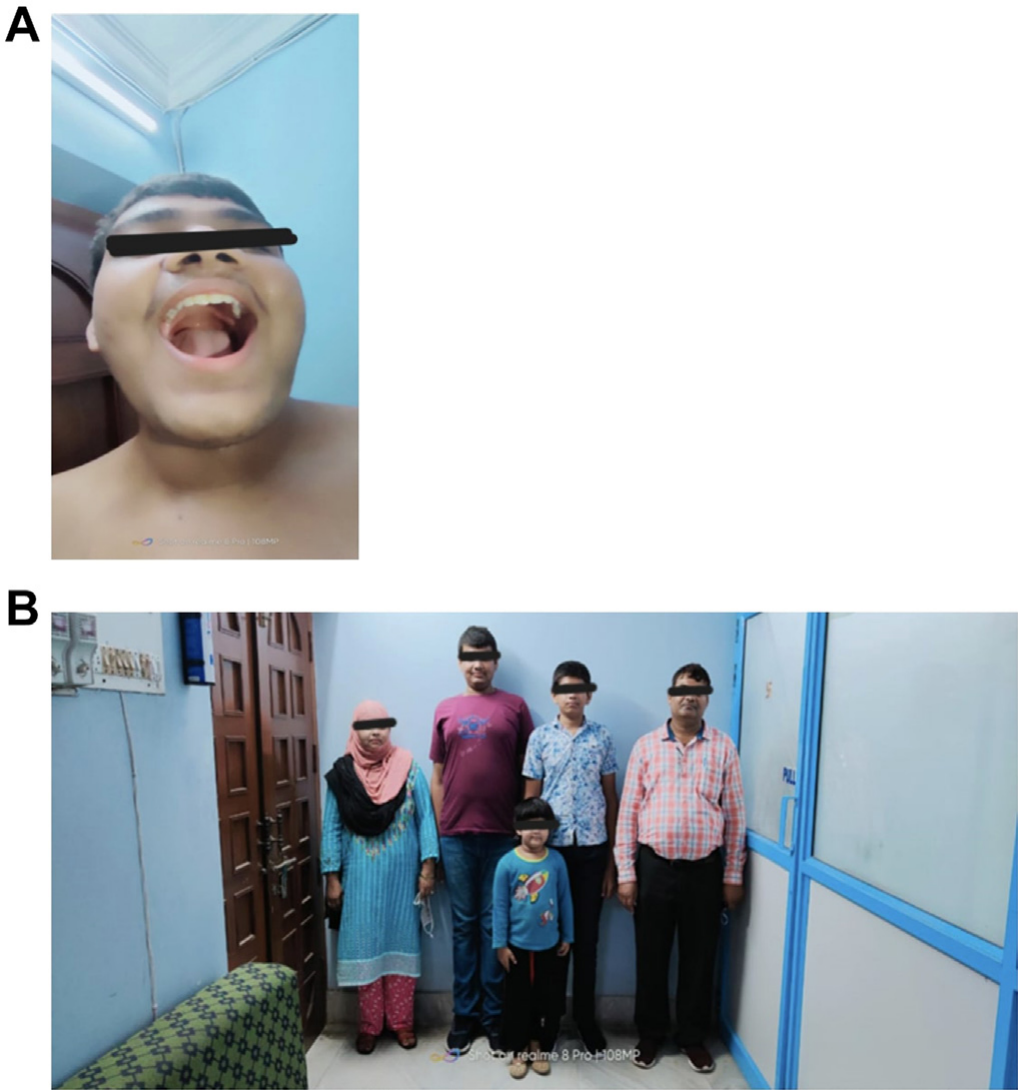
*A*: Showing high arch palate, and *B*: Photograph of the family showing from *left* to *right*: carrier mother, index case, noncarrier sister, brother of the index case who has homozygous mutation and carrier father.

**Fig. 2:**
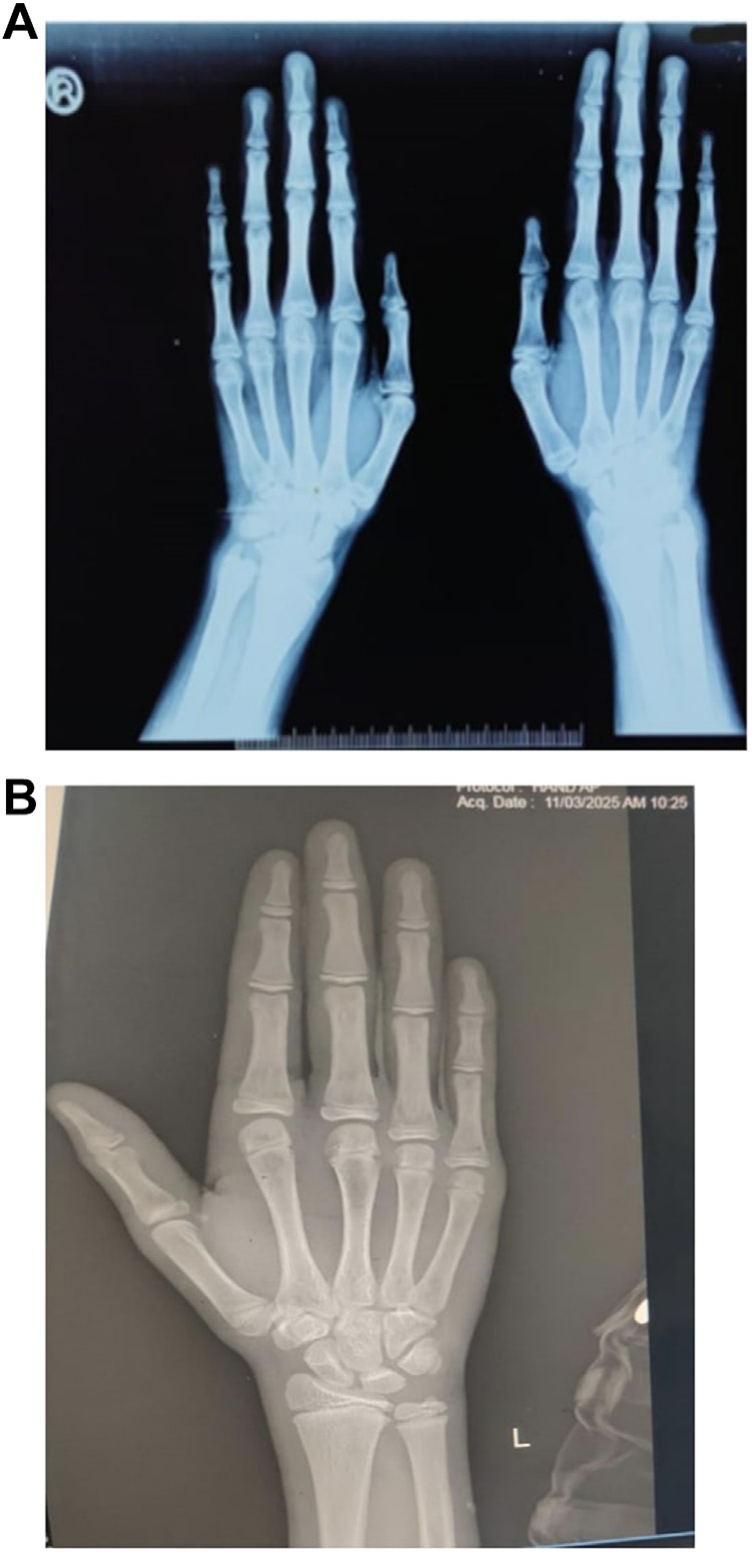
*A*: x-ray of the hand showing ARACHNODACTYLY, *B*: Normal x-ray of the hand of a 17-y-old male.

Upon examination, his height was noted to be 178 cm, placing him in the 90th percentile for his age, with a mid-parental height of 164 cm (10th percentile) (Table). His upper segment to lower segment ratio was calculated at 0.85. Physical features included a high arch palate, arachnodactyly, chest wall deformity, scoliosis, and genu valgum, though his head size and facial features appeared normal (Figs. 1 *A* and 2). Acanthosis nigricans were observed on his skin, but his blood pressure and pulse rate were within normal ranges. His pubic hair was P4, and testicular volume of 15 ml. Intellectual assessment yielded an Intelligence Quotient of 58. His ocular and cardiovascular examinations did not reveal any abnormalities, and his reproductive development was noted as normal.

**Table:** Anthropometric and Biochemical Features of the Family

Individual	Height (cm)	Arm span (cm)	Arm span–Height (cm)	Upper segment (cm)	Upper-to-lower segment ratio	Weight (kg)	BMI (kg/m^2^)	Homocysteine (μmol/L)	Reference range (μmol/L)
Proband	178	198	+20	82	0.85	86	27.14	199.95 → 225.04 (post-pyridoxine)	5.45–16.2
Affected brother	165	179	+14	74	0.81	58.6	21.41	166.00	5.45–16.2
Father	166	177.5	+12.5	-	-	-	-	25.78	5.46–16.2
Mother	149	152	+3	-	-	-	-	7.69	4.44–13.56
Sister	110	114	+4	-	-	-	-	6.54	Non-folate suppl.: < 10
									Folate

Abbreviations: BMI = body mass index.

Family anthropometrics revealed normal stature for the parents and sister. However, the proband’s 13-year-old brother also exhibited tall stature (height: 165 cm, arm span: 179 cm, arm span-to-height ratio: +14 cm) and skeletal anomalies (upper-to-lower segment ratio: 0.81), similar to the proband (Fig. 1 *B*). Neither the proband nor the brother had ocular abnormalities, but the brother did not have a history of seizures, unlike the proband. The father, a heterozygous carrier, had an increased arm span-to-height ratio (+12.5 cm) and mildly elevated homocysteine levels (25.78 μmol/L), but no other clinical manifestations of homocystinuria. This familial pattern, with shared skeletal features in the proband, affected brother, and carrier father, strongly suggested a genetic or syndromic cause, likely homocystinuria (Table).

Diagnostic tests revealed normal growth hormone levels, HbA1c, serum vitamin B12 and folate levels. The proband had a low 25-OH-vitamin D level of 6.1 ng/mL (20–40 ng/mL), indicating vitamin D deficiency, which was addressed with supplementation. However, his serum homocysteine level was significantly elevated at 199.95 μmol/L (5.45-16.2 μmol/L), which increased further to 225.04 μmol/L after pyridoxine administration, indicating a non-response to this treatment. His younger brother also showed elevated homocysteine levels. Genetic sequencing identified a homozygous missense variant in exon 12 of the MTHFR gene (chr1:g.11791208T>G; depth: 121x), resulting in the amino acid substitution of threonine for lysine at codon 625 (p.Lys625Thr; ENST00000376585.6). In silico predictions suggest a damaging effect on protein function, as indicated by PolyPhen-2 (HumDiv: possibly damaging), SIFT, LRT, and MutationTaster2 (all classified as damaging). Additionally, the reference codon is highly conserved across species, further supporting its functional significance.This variant was classified as likely pathogenic based on multiple lines of evidence. It has not been reported in the 1000 genomes project, gnomAD, or internal databases, indicating its rarity.^4^^,^^5^

Segregation analysis confirmed that the variant was homozygous in the case and his affected brother, while the unaffected sister was not a carrier. Testing conducted in both parents were heterozygous carriers of this variant, consistent with autosomal recessive inheritance.

His management included a low-methionine diet with protein intake restricted, tailored to his nutritional needs. He was prescribed pyridoxine (vitamin B6) at a dose of 210 mg/d, divided into morning and evening doses. Additionally, he received a daily supplement containing cyanocobalamin 400 mcg, folic acid 1 mg, and vitamin B6 5 mg. Due to the lack of response to pyridoxine therapy, treatment with betaine was initiated at a dose of 2 g three times daily (6 g/d), alongside his ongoing antiepileptic medication. Plasma homocysteine levels were regularly monitored to guide treatment adjustments.

## Discussion

We report a case of a 17-year-old male presenting with tall stature, intellectual disability, and skeletal abnormalities, diagnosed with a novel variant in the *MTHFR* gene. Interestingly, the carrier father exhibited a subtle phenotypic feature—a greater arm span than height—mirroring the proband's skeletal finding. This emphasizes the potential for subtle traits in carriers. To our knowledge, this is the first reported case highlighting an increased arm span-to-height ratio as a subtle phenotypic feature in a carrier of homocystinuria. This observation underscores the importance of assessing carrier phenotypes during genetic evaluations, as such findings can provide critical diagnostic insights.

The proband showed no response to pyridoxine, with paradoxically increased homocysteine levels, necessitating a shift to betaine therapy. This lack of response indicates that the homozygous missense variant in the *MTHFR* gene disrupts remethylation pathways rather than the transsulfuration pathway, where pyridoxine (vitamin B6) enhances cystathionine β-synthase activity.^6^ Pyridoxine is effective in pyridoxine-responsive homocystinuria, where cystathionine β-synthase retains partial activity, but it is ineffective in cases like this, where the defect lies in the remethylation pathway.^7^ Genetic testing was critical in guiding the shift to betaine therapy, an alternative methyl donor that bypasses the dysfunctional enzyme pathway. Betaine acts by remethylating homocysteine to methionine via the enzyme betaine-homocysteine methyltransferase, providing an effective treatment for pyridoxine-unresponsive homocystinuria.^8^ This case highlights the complexity of managing novel genetic variants and their unpredictable treatment responses.

While typically asymptomatic, carrier individuals of certain genetic disorders may exhibit subtle phenotypic features that provide valuable diagnostic clues. In this case, the carrier father demonstrated a subtle phenotypic feature—a greater arm span than height—alongside a moderately elevated homocysteine level of 25.78 μmol/L (carriers might have slightly raised levels), compared to the mother’s level of 7.69 μmol/L.^9^ To our knowledge, this is the first reported case highlighting an increased arm span-to-height ratio as a subtle phenotypic feature in a carrier of homocystinuria. This observation underscores the importance of assessing carrier phenotypes during genetic evaluations, as such findings can provide critical diagnostic insights. Analogous findings have been reported in other genetic conditions. For example, carriers of Duchenne muscular dystrophy may experience mild muscle weakness due to partial dystrophin deficiency, while carriers of hemophilia can exhibit prolonged bleeding tendencies due to suboptimal clotting factor levels.^10^^,^^11^ These examples highlight the potential for carriers of recessive conditions to present with identifiable physical or biochemical traits, even when they do not manifest the full disease phenotype.

An intriguing aspect of this case is the phenotypic disparity observed between the carrier father and mother. The father exhibited a greater arm span-to-height ratio and mildly elevated homocysteine levels (25.78 μmol/L), while the mother showed no such features. This variability may be influenced by hormonal factors, such as the protective role of estrogen in females, which enhances methionine metabolism and lowers homocysteine levels.^12^ In contrast, testosterone in males may contribute to less efficient homocysteine metabolism, potentially exacerbating subtle phenotypic traits.^13^ Additionally, genetic modifiers and X-inactivation in females could further modulate phenotypic expression.^14^ Similar sex-based differences have been observed in other conditions, such as hereditary hemochromatosis, where males are more likely to exhibit subtle symptoms due to differences in iron metabolism.^15^ These findings underscore the complexity of phenotypic variability in carriers of autosomal recessive disorders and highlight the need for further research. Exploring the interplay of hormonal, genetic, and epigenetic factors could enhance our understanding of disease mechanisms and provide more effective tools for clinical diagnosis and management. This case also emphasizes the importance of detailed phenotypic assessments, even in carriers, as they may reveal valuable diagnostic insights that aid in understanding the broader implications of genetic mutations.

This case illustrates the need for regular biochemical monitoring to assess treatment efficacy and adjust therapy as needed. It also highlights the importance of genetic studies in identifying novel mutations and informing management strategies, particularly in rare disorders like homocystinuria. Homocystinuria is a significant cause of syndromic tall stature, requiring comprehensive evaluation and management. Identifying a novel genetic variant associated with this condition underscores the ongoing need for genetic research and its implications for diagnosis, treatment, and family screening. The subtle phenotypic presentations in carriers highlight the importance of detailed family histories and genetic evaluations in diagnosing rare genetic disorders.

## Patient Consent

Obtained

## Disclosure

The authors have no conflicts of interest to disclose.
